# The different dietary sugars modulate the composition of the gut microbiota in honeybee during overwintering

**DOI:** 10.1186/s12866-020-01726-6

**Published:** 2020-03-17

**Authors:** Hongfang Wang, Chunlei Liu, Zhenguo Liu, Ying Wang, Lanting Ma, Baohua Xu

**Affiliations:** College of Animal Science and Technology, Shandong Agricultural University, Tai’an, 271018 Shandong China

**Keywords:** Honeybees, Sugar diet, Gut bacteria, Microbial diversity, Overwintering

## Abstract

**Background:**

The health of honeybee colonies is critical for bee products and agricultural production, and colony health is closely associated with the bacteria in the guts of honeybees. Although colony loss in winter is now the primary restriction in beekeeping, the effects of different sugars as winter food on the health of honeybee colonies are not well understood. Therefore, in this study, the influence of different sugar diets on honeybee gut bacteria during overwintering was examined.

**Results:**

The bacterial communities in honeybee midguts and hindguts before winter and after bees were fed honey, sucrose, and high-fructose syrup as winter-food were determined by targeting the V3-V4 region of 16S rDNA using the Illumina MiSeq platform.

The dominant microbiota in honeybee guts were the phyla Proteobacteria (63.17%), Firmicutes (17.61%; *Lactobacillus*, 15.91%), Actinobacteria (4.06%; *Bifidobacterium*, 3.34%), and Bacteroidetes (1.72%). The dominant taxa were conserved and not affected by season, type of overwintering sugar, or spatial position in the gut. However, the relative abundance of the dominant taxa was affected by those factors. In the midgut, microbial diversity of the sucrose group was higher than that of the honey and high-fructose syrup groups, but in the hindgut, microbial diversity of the honey and high-fructose groups was higher than that in the sucrose group. Sucrose increased the relative abundance of Actinobacteria (Bifidobacteriales *Bifidobacteriaceae*) and Alphaproteobacteria (Rhizobiales and *Mitochondria*) of honeybee midgut, and honey enriched the Bacteroidetes and Gammaproteobacteria (Pasteurellales) in honeybee hindgut. High-fructose syrup increased the relative abundance of Betaproteobacteria (Neisseriales: *Neisseriaceae*) of the midgut.

**Conclusion:**

The type of sugar used as winter food affected the relative abundance of the dominant bacterial communities in honeybee guts, not the taxa, which could affect the health and safety of honeybee colonies during overwintering. The presence of the supernal Alphaproteobacteria, Bifidobacteriales, and *Lactobacillaceae* in the gut of honeybees fed sucrose and cheaper than honey both indicate that sucrose is very suitable as the overwintering food for honeybees.

## Background

Honeybees (*Apis mellifera*) are important crop pollinators used extensively in agriculture and food production worldwide [1]. Microbes are highly abundant in the gut of honeybees, and gut microbial communities can impact bee pollinators in diverse ways, from nutrition to defense against disease [2, 3]. Gut microbes are important in bee health and disease [4–7]. For example, *Lactobacillus* and *Acetobacter aceti* can increase honeybee immunity and protect against pathogenic bacteria [4, 8]. The honeybee is a social insect that harbors a core gut microbiota of nine abundant phylotypes, which account for 95% of all gut bacteria [9, 10]. Adult honeybees have a distinct microbial gut community comprised of 9 core bacterial species in the Alphaprotobacteria, Betaprotobacteria, Gammaprotobacteria, Firmicutes and Actinobacteria [11, 12]. However, the composition of the honeybee gut microbial community is dynamic, not fixed. For example, gut microbes provide different advantages in honeybees of different ages (measured in days) [13]. In addition, gut microbiota are transmitted from adults to newly hatched bees through feeding and secretion inside the colony [14]. The major changes in the composition of honeybee midgut/pyloric microbiota throughout one foraging season have been documented, and the composition of the community remained stable under stable environmental conditions during winter [15]. Furthermore, intestinal microbiota of honeybee varied with various niche of digestive tract. There are few bacterial phylotypes and low abundance in midgut (1–4%), but richer phylotypes and higher abundance in hindgut (> 90%) [12]. Gammaproteobacteria and Beta Gammaproteobacteria are the dominant taxa in the midgut [15]. In addition to Gammaproteobacteria and Betaproteobacteria, the dominant microbiota in the hindgut also include fermentative bacteria used for fermenting food residues and faeces, such as *lactobacillu*s [12, 13, 16].

Although affected by age, season, temperature and niche, among other factors, the response of honeybee gut microbiota to different diets during winter remains unclear. Honey is the primary natural food for honeybees in winter, but the usual foods in beekeeping are sucrose and high-fructose syrup, because they are less expensive. However, whether sucrose and high-fructose syrup are suitable for feeding honeybees in winter remains to be evaluated. In this study, to evaluate the suitability and safety of different sugars, the influence of honey, sucrose, and high-fructose syrup on the midgut and hindgut microbiota of honeybees during winter was determined by using high-throughput 16S rRNA gene sequencing.

## Results

### Honeybee gut microbiota profile

In this study, the gut bacterial community of honeybees was characterized via 16S rRNA amplicon Illumina sequencing. A quality control procedure was performed on the paired-end reads, including trimming the barcodes and primers and filtering low-quality reads and chimeras, according to QIIME to yield the results for processed data (Table S1). As shown in Fig. S1, the rarefaction curves for all samples approached a saturation plateau, indicating that the current analysis contained adequate depth to capture most microbial diversity information.

**Fig. 1:**
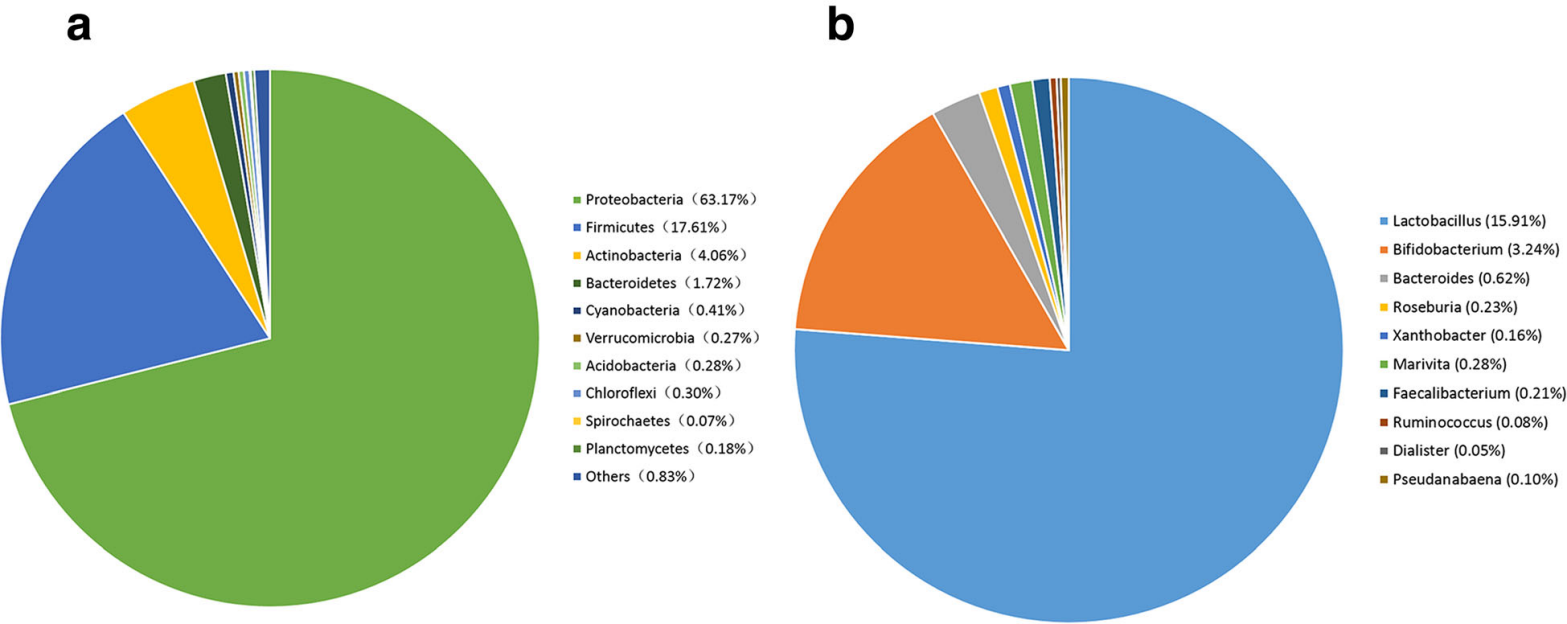
Composition of the top 10 intestinal bacteria. **a**: Composition of the top 10 intestinal bacteria at the level of phylum. **b**: Composition of the top 10 intestinal bacteria at the level of genus

All valid reads were classified taxonomically (phylum, class, order, family, and genus levels) using QIIME (Table S3). The top 10 most abundant microbes in honeybee guts are presented in Fig. S2. The top 10 most abundant phyla in the honeybee gut were Proteobacteria, Firmicutes, Actinobacteria, Bacteroidetes, Cyanobacteria, Verrucomicrobia, Acidobacteria, Chloroflexi, Spirochaetes, and Planctomycetes (Fig. S2A). The top 10 most abundant genera of bacteria in the honeybee gut were *Lactobacillus*, *Bifidobacterium*, *Bacteroides*, *Roseburia*, *Xanthobacter*, *Marivita*, *Faecalibacterium*, *Ruminococcus*, *Dialister*, and *Pseudanabaena* (Fig. S2E). When the data from the midgut and hindgut were combined, the dominant populations were Proteobacteria (63.17%), Firmicutes (17.61%), Actinobacteria (4.06%), and Bacteroidetes (1.72%) at the phylum level (Fig. 1a) and *Lactobacillus* (15.91%) and *Bifidobacterium* (3.24%) at the genus level (Fig. 1b), regardless of intestinal segment or sugar type.

**Fig. 2:**
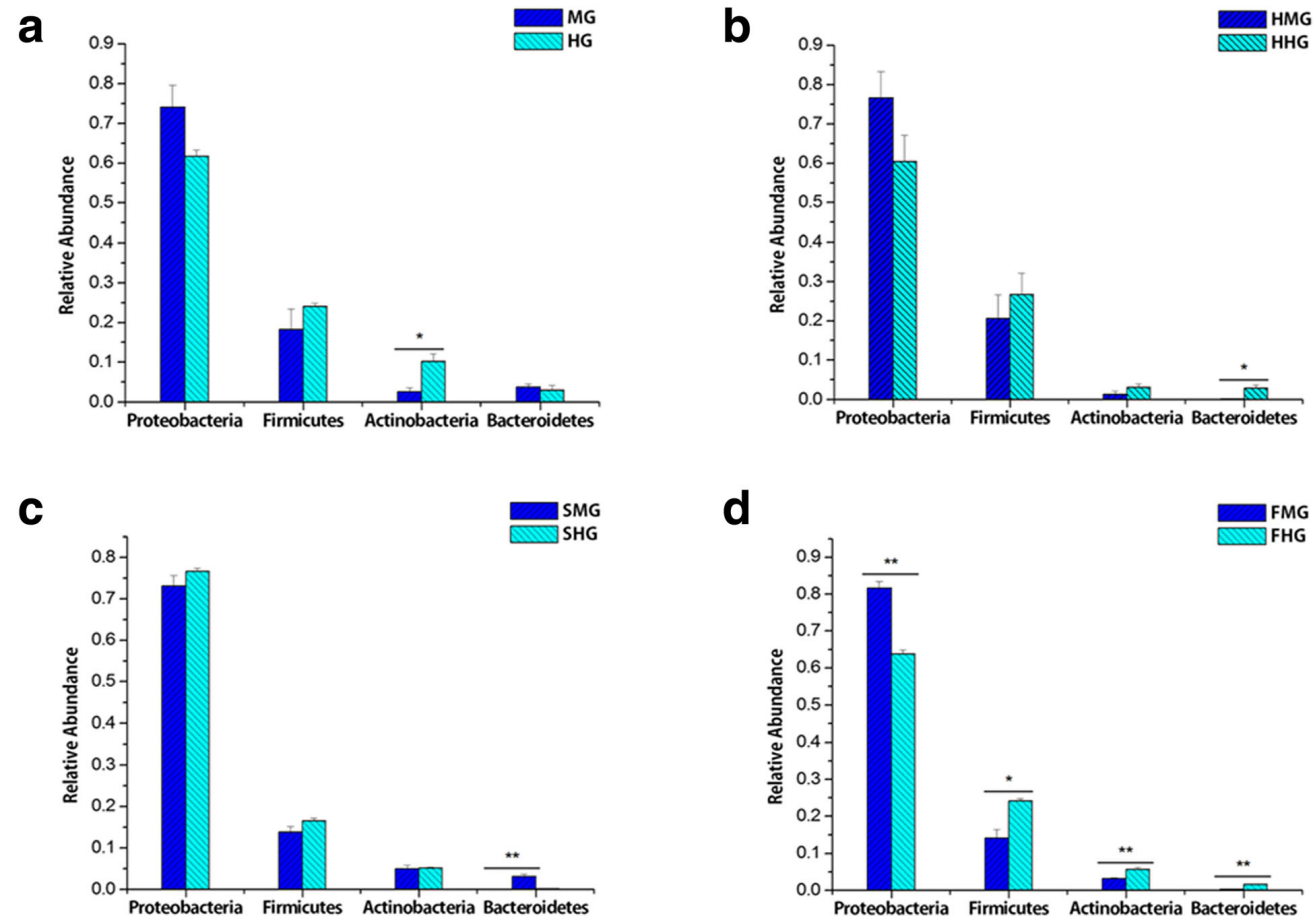
Differences in the dominant bacteria at the level of phylum between the midgut and hindgut of honeybees. MG, midgut sample from pre-overwintering honeybees; SMG, midgut sample after honeybees were fed sucrose as the overwintering food; FMG, midgut sample after bees were fed high-fructose syrup as the overwintering food; HMG, midgut sample after bees were fed honey as the overwintering food; HG, hindgut sample from pre-overwintering honeybees; SHG, hindgut sample after bees were fed sucrose as the overwintering food; FHG, hindgut sample after bees were fed high-fructose syrup as the overwintering food; HHG, hindgut sample after bees were fed honey as the overwintering food (the abbreviations represent the same in the following figures). The column marked with “**” shows extremely significant differences (*P* < 0.01), and the column marked with “*” shows significant differences (*P* < 0.05)

Based on further analysis of the dominant microbiota at the phylum and genus levels in the honeybee gut, the dominant taxa were not different between the midgut and hindgut. However, the relative abundance of these groups was different between midgut and hindgut and was affected by sugar type. Before overwinter feeding, the abundance of Actinobacteria in the hindgut was significantly higher than that in the midgut (*P* < 0.05, Fig. 2a), but this distinction disappeared after the bees were fed honey and sucrose. The difference in abundance of Bacteroidetes between midgut and hindgut was also affected by sugar type. Before overwinter feeding, the abundance of Bacteroidetes was not significantly different between midgut and hindgut (*P* > 0.05). However, the relative abundance of Bacteroidetes in the hindgut was significantly higher than that in the midgut after the bees were fed honey (*P* < 0.05, Fig. 2b) and high-fructose syrup (*P* < 0.01, Fig. 2d) for overwintering. In general, the abundance of most dominant bacterial taxa was higher in the hindgut than in the midgut. The sucrose group was an exception, and the relative abundance of Bacteroidetes in the hindgut was significantly lower than that in the midgut (*P* < 0.01, Fig. 2c).

**Fig. 3:**
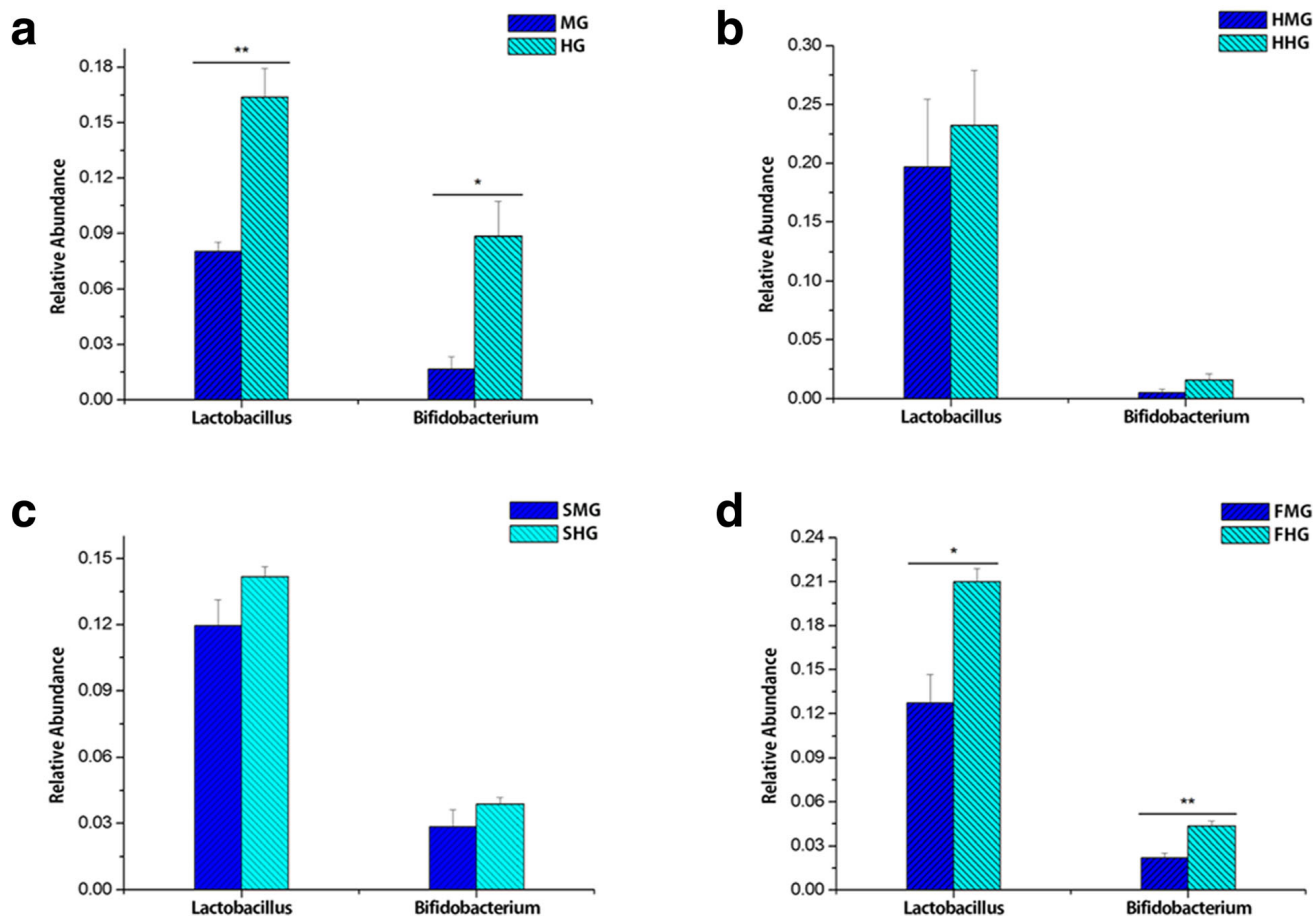
Differences in the dominant bacteria at the level of genus between the midgut and hindgut of honeybees. The column marked with “**” shows extremely significant differences (*P* < 0.01), and the column marked with “*” shows significant differences (*P* < 0.05)

For the dominant genera of bacteria, the relative abundance of *Lactobacillus* and *Bifidobacterium* was significantly higher in the hindgut than in the midgut during prewinter and in the high-fructose syrup group (*P* < 0.05, Fig. 3 ad). However, the difference in abundance of *Lactobacillus* and *Bifidobacterium* between the midgut and hindgut disappeared after the bees were fed honey and sucrose for overwintering *(P* > 0.05, Fig. 3 bc). In general, the abundance of most dominant microbiota was higher in the hindgut than in the midgut.

### The differences in gut microbiota of honeybees fed honey, sucrose, and high-fructose syrup

**Fig. 4:**
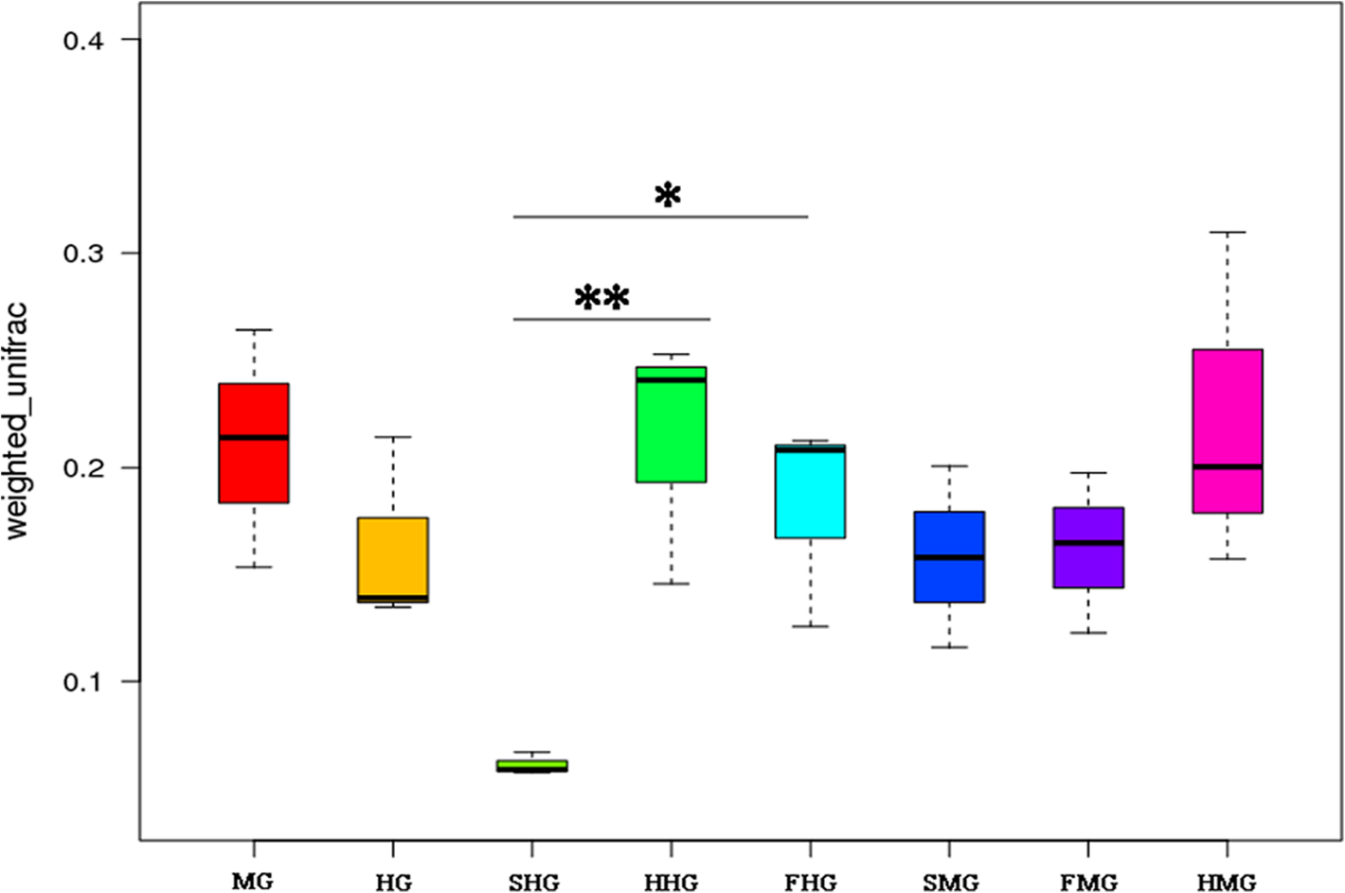
Diversity of honeybee intestinal bacteria based on the weighted unifrac beta diversity analysis

The microbial diversity in the midgut and hindgut of honeybees fed different types of sugars as winter food was analyzed using beta-diversity analysis. The microbial diversity of honeybee midguts was not significantly different among the honey, sucrose, and high-fructose syrup groups (*P* > 0.05, Fig. 4; Table S2). However, the microbial diversity of honeybee hindguts was affected by sugar type, with higher diversity in the hindguts of honeybees fed honey and high-fructose syrup than in those fed sucrose (*P* < 0.05, Fig. 4; Table S2).

**Fig. 5:**
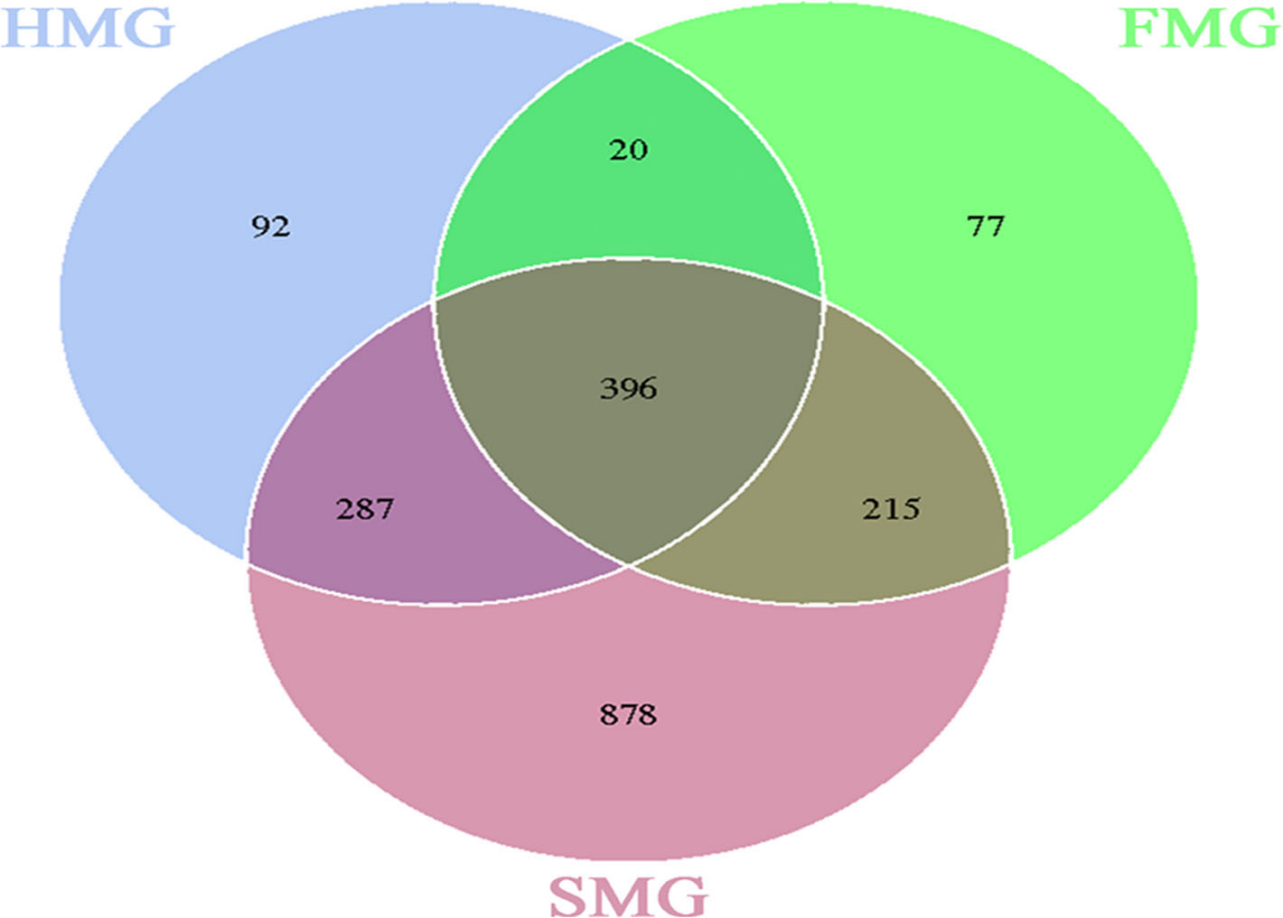
Numbers of the midgut OTUs in the three sugar groups

**Fig. 6:**
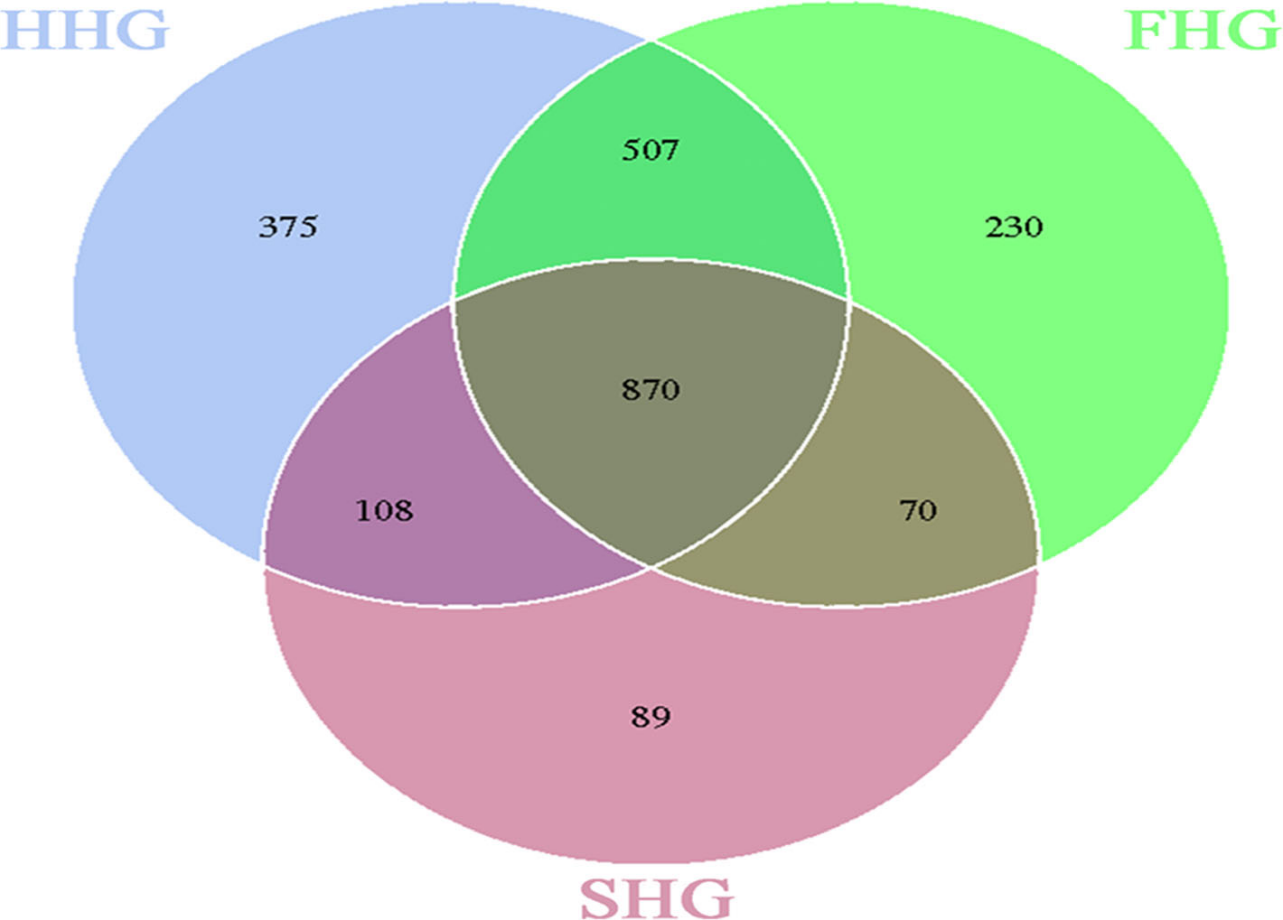
Numbers of the hindgut OTUs in the three sugar groups

From the clustering analysis of OTUs, Venn diagrams were created to analyze the common and unique OTUs among the three sugar groups. Among the three groups, 396 OTUs were common to the midguts (Fig. 5), and 870 OTUs were common to the hindguts (Fig. 6). As shown in Fig. 5, 878 OTUs were unique to the midgut of the sucrose group, which was more than in the honey (92 OTUs) and the high-fructose syrup (77 OTUs) groups. However, in the hindgut, only 89 OTUs were unique in the sucrose group, far less than in the honey (375 OTUs) and high-fructose syrup (230 OTUs) groups (Fig. 6). Thus, in the midgut, the bacterial diversity of the sucrose group was higher than that in the honey and high-fructose syrup groups, whereas in the hindgut, the bacterial diversity of the honey and high-fructose groups was higher than that in the sucrose group.

**Fig. 7:**
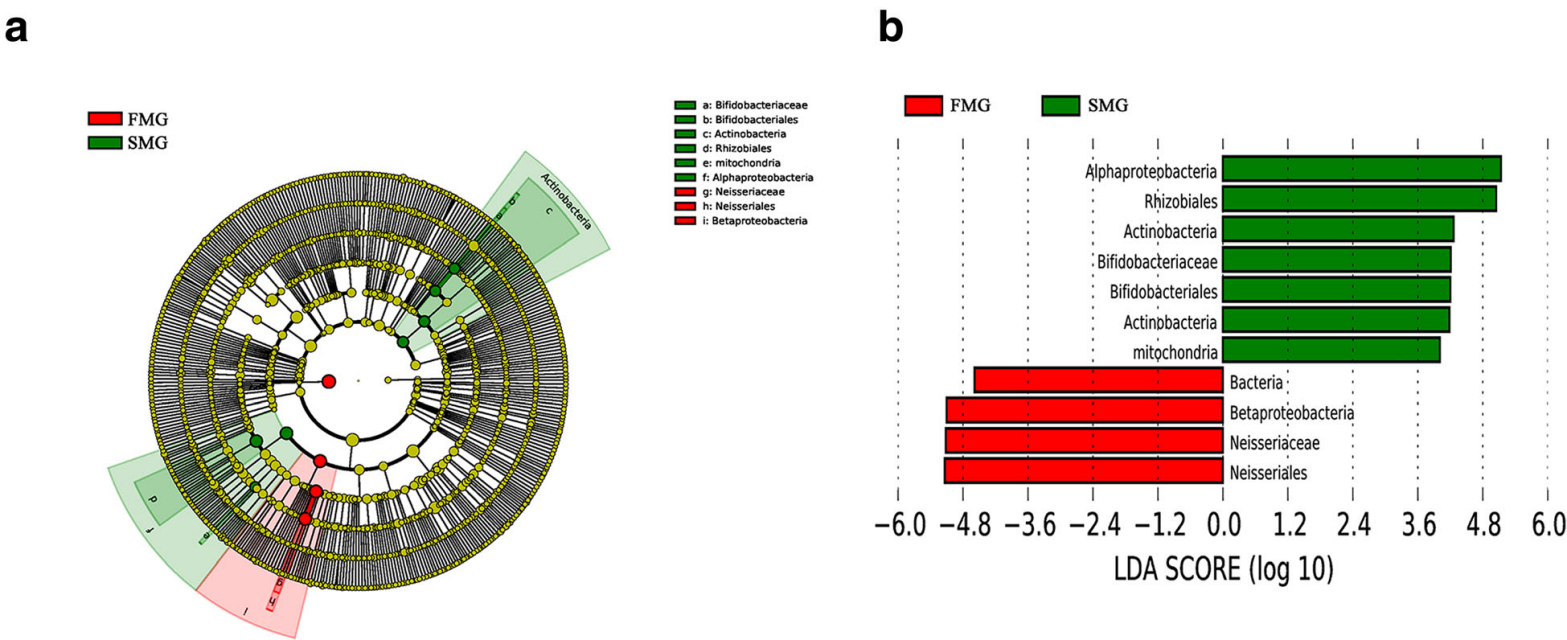
The main taxa of bacteria that were different in HMG vs. SMG vs. FMG. **a**: Cladogram of the main taxa of microbiota that were different on the basis of LEfSe analysis. **b**: LEfSe analysis (taxa with LDA score > 4). Color code: Yellow (A) represents no significant difference in taxa; Green (A and B) represents significantly different taxa, with their highest relative abundance in SMG; Red (A and B) represents significantly different taxa, with their highest relative abundance in FMG

**Fig. 8:**
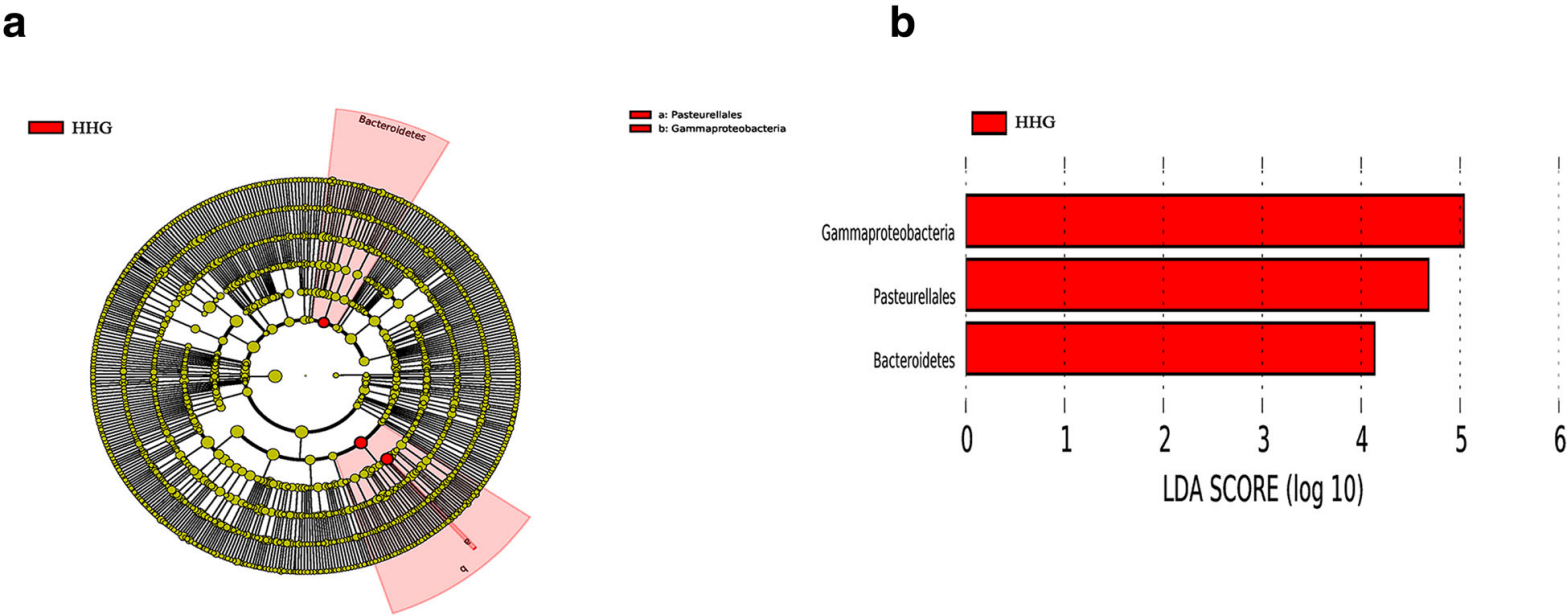
The main taxa of bacteria that were different in HHG vs. SHG vs. FHG. **a**: Cladogram of the main taxa of microbiota that were different on the basis of LEfSe analysis. **b**: LEfSe analysis (taxa with LDA score > 4). Color code: Red represents significantly different taxa, with their highest relative abundance in HHG

The detailed changes in the gut microorganisms of bees fed the different sugar types were analyzed using LEfSe. The contributions of the microorganisms to the differences among the groups were evaluated using the LDA score. The results for the midgut comparisons (HMG vs. FMG vs. SMG) are shown in Fig. 7, and the main taxa that were different were Betaproteobacteria (Neisseriales: *Neisseriaceae*), Actinobacteria (Bifidobacteriales: *Bifidobacteriaceae*), and Alphaproteobacteria (Rhizobiales and *Mitochondria*). The relative abundance of Betaproteobacteria (Neisseriales: *Neisseriaceae*) was the highest in the high-fructose syrup group, whereas in the bees fed sucrose, the relative abundance of Actinobacteria (Bifidobacteriales *Bifidobacteriaceae*) and Alphaproteobacteria (Rhizobiales and *Mitochondria*) increased. In the hindgut comparisons, the main taxa that were different among the three groups were the Bacteroidetes and Gammaproteobacteria (Pasteurellales) (Fig. 8), which increased in the honey group.

### The differences in honeybee gut microbiota between prewinter and postfeeding for overwintering

**Fig. 9:**
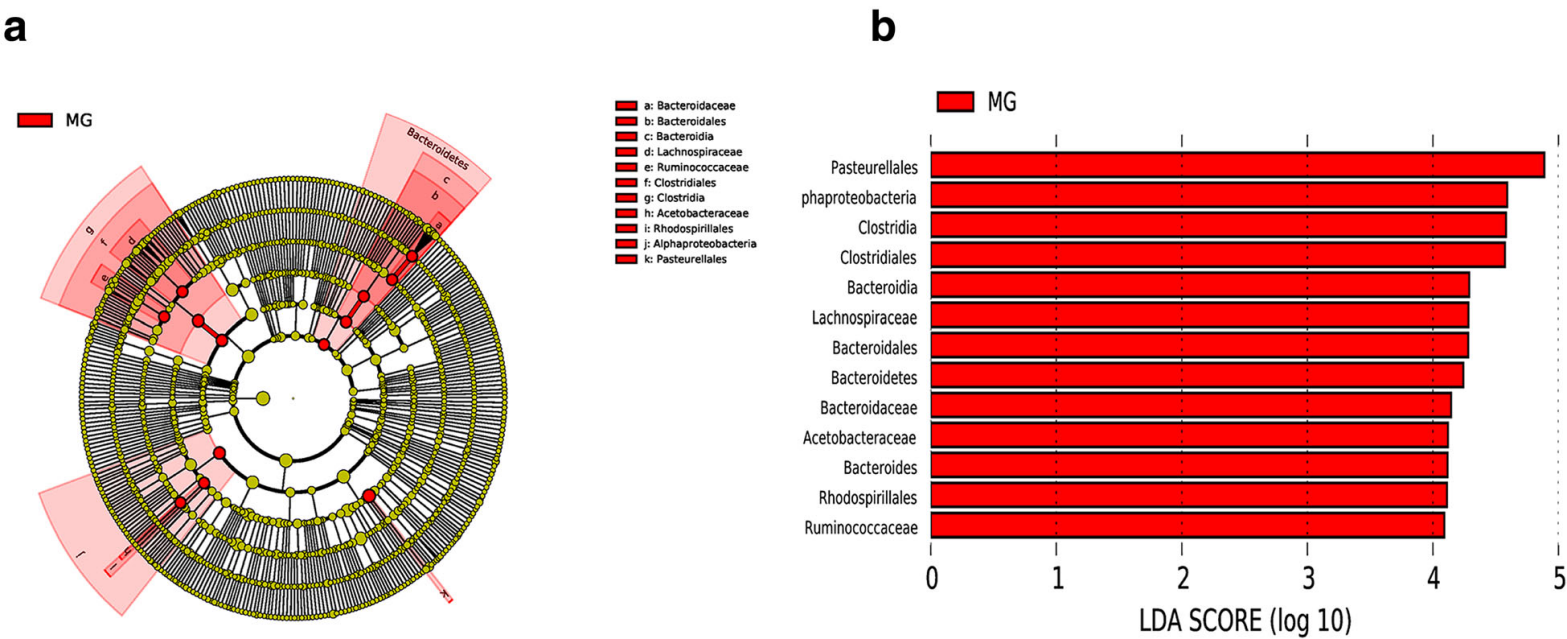
The main taxa of bacteria that were different in MG vs. HMG. **a**: Cladogram of the main taxa of microbiota that were different on the basis of LEfSe analysis. **b**: LEfSe analysis (taxa with LDA score > 4). Color code: Red represents significantly different taxa, with their highest relative abundance in MG

**Fig. 10:**
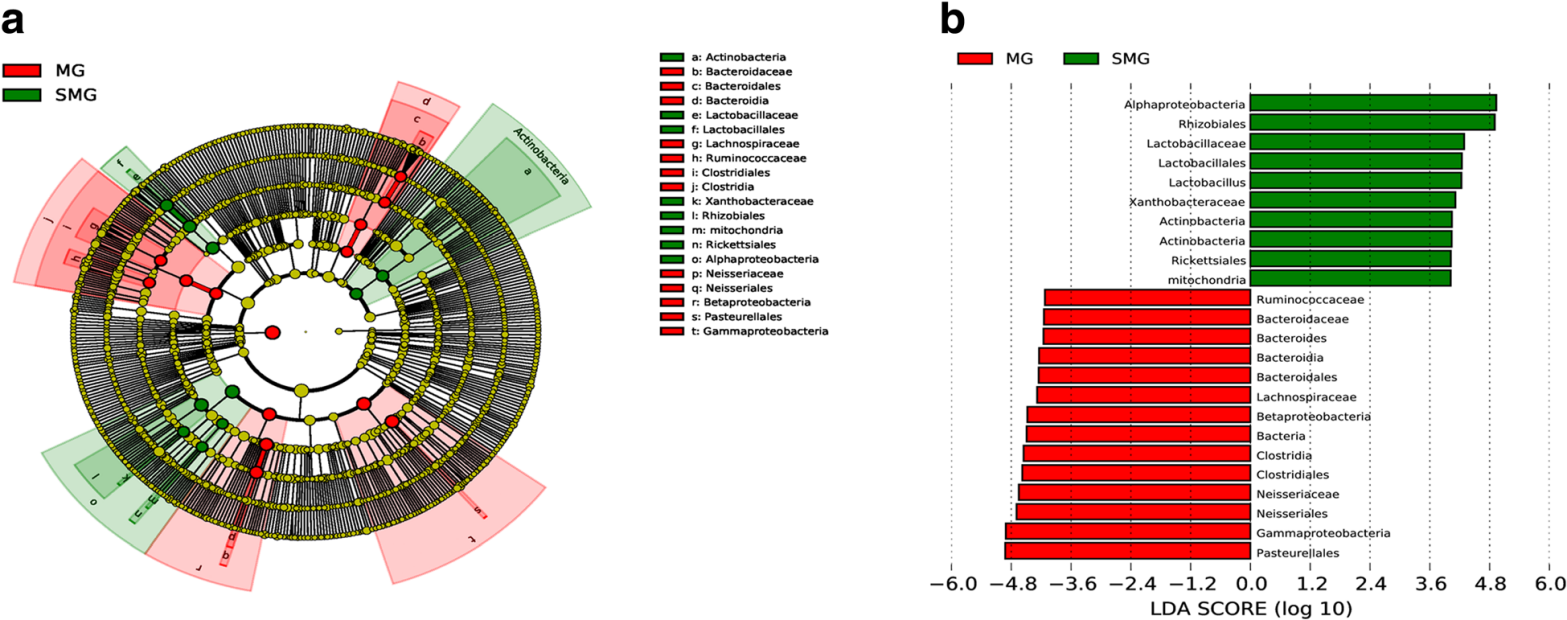
The main taxa of bacteria that were different in MG vs. SMG. **a**: Cladogram of the main taxa of bacteria that were different on the basis of LEfSe analysis. **b**: LEfSe analysis (taxa with LDA score > 4). Color code: Red represents significantly different taxa, with their highest relative abundance in MG; Green represents significantly different taxa, with their highest relative abundance in SMG

**Fig. 11:**
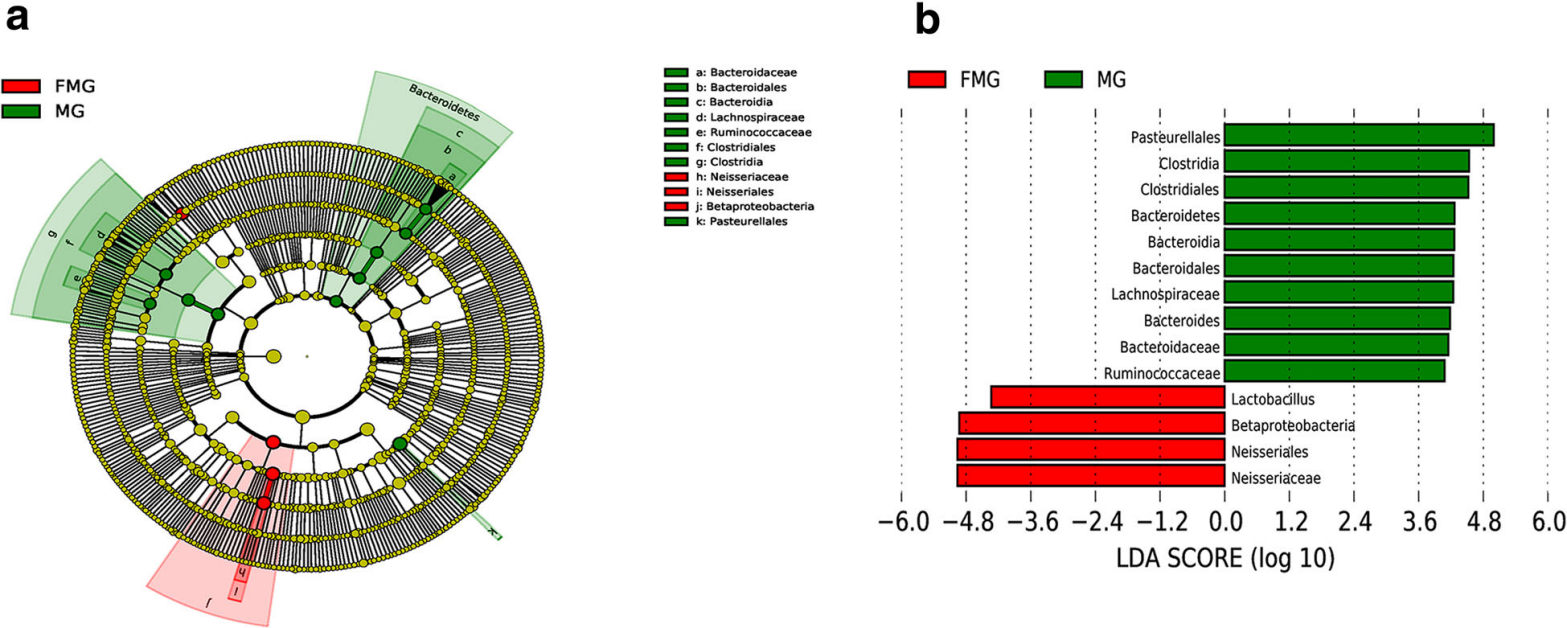
The main taxa of bacteria that were different in MG vs. FMG. **a**: Cladogram of the main taxa of microflora that were different on the basis of LEfSe analysis. **b**: LEfSe analysis (taxa with LDA score > 4). Color code: Green represents significantly different taxa, with their highest relative abundance in MG; Red represents significantly different taxa, with their highest relative abundance in FMG

The differences in gut microbes were analyzed between preoverwintering and postfeeding overwintering bees. The primary taxa that were different in the midguts of honeybees between prewinter and postfeeding for overwintering included Bacteroidetes, Clostridia (*Lachnospiraceae* and *Ruminococcaeae*), and Pasteurellales, independent of sugar type (Figs. 9, 10, 11). However, some sugar-specific bacterial taxa were identified in midguts between prewinter and postfeeding for overwintering bees (e.g., MG vs. HMG: Rhodospirillales *Acetobacteraceae* of Alphaproteobacteria was more abundant in MG; MG vs. SMG/FMG: *Neisseriaceae* of Betaproteobacteria was more prominent in MG). In addition, compared with MG, sucrose as the winter food increased the colonization of Actinobacteria, Lactobacillales (*Lactobacillaceae*), and Alphaproteobacteria (*Xanthobacteraceae*, Rickettsiales and *Mitochondria*) in the midgut of honeybees (Fig. 10).

**Fig. 12:**
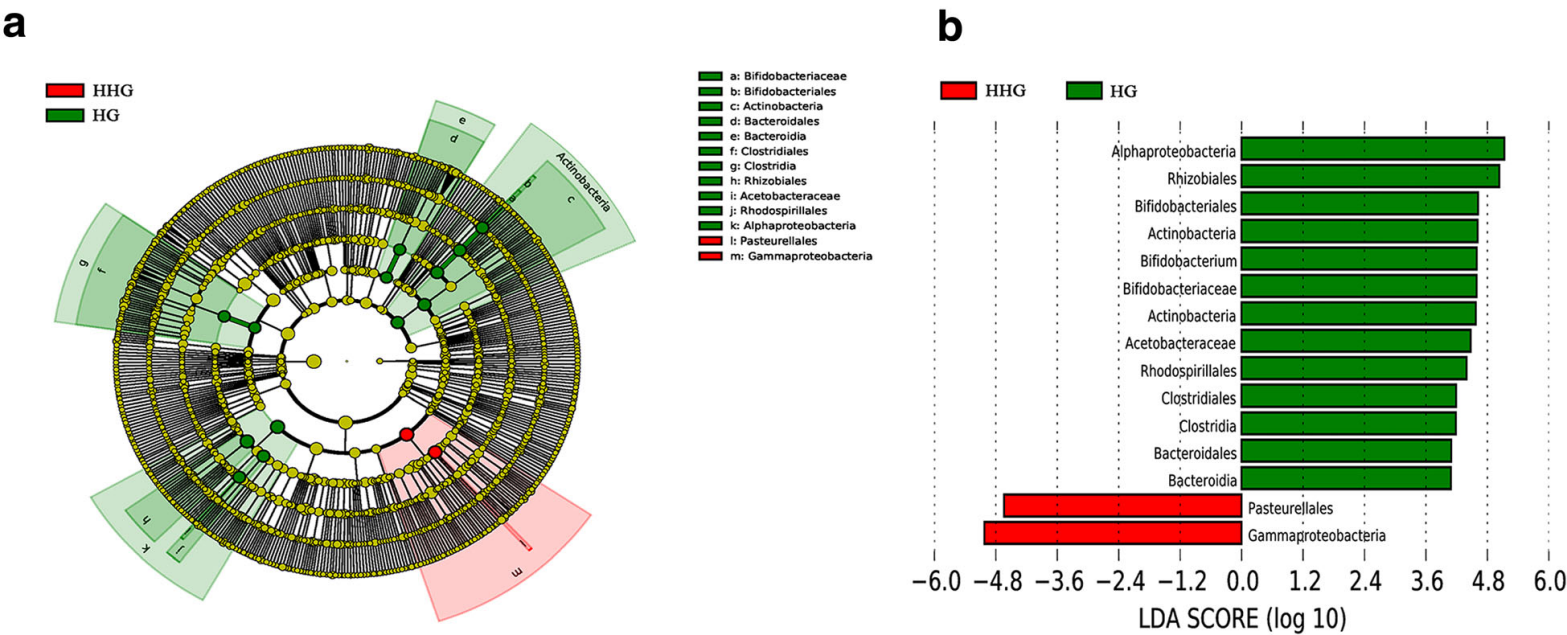
The main taxa of bacteria that were different in HG vs. HHG. **a**: Cladogram of the main taxa of micrflora that were different on the basis of LEfSe analysis. **b**: LEfSe analysis (taxa with LDA score > 4). Color code: Green represents significantly different taxa, with their highest relative abundance in HG; Red represents significantly different taxa, with their highest relative abundance in HHG

**Fig. 13:**
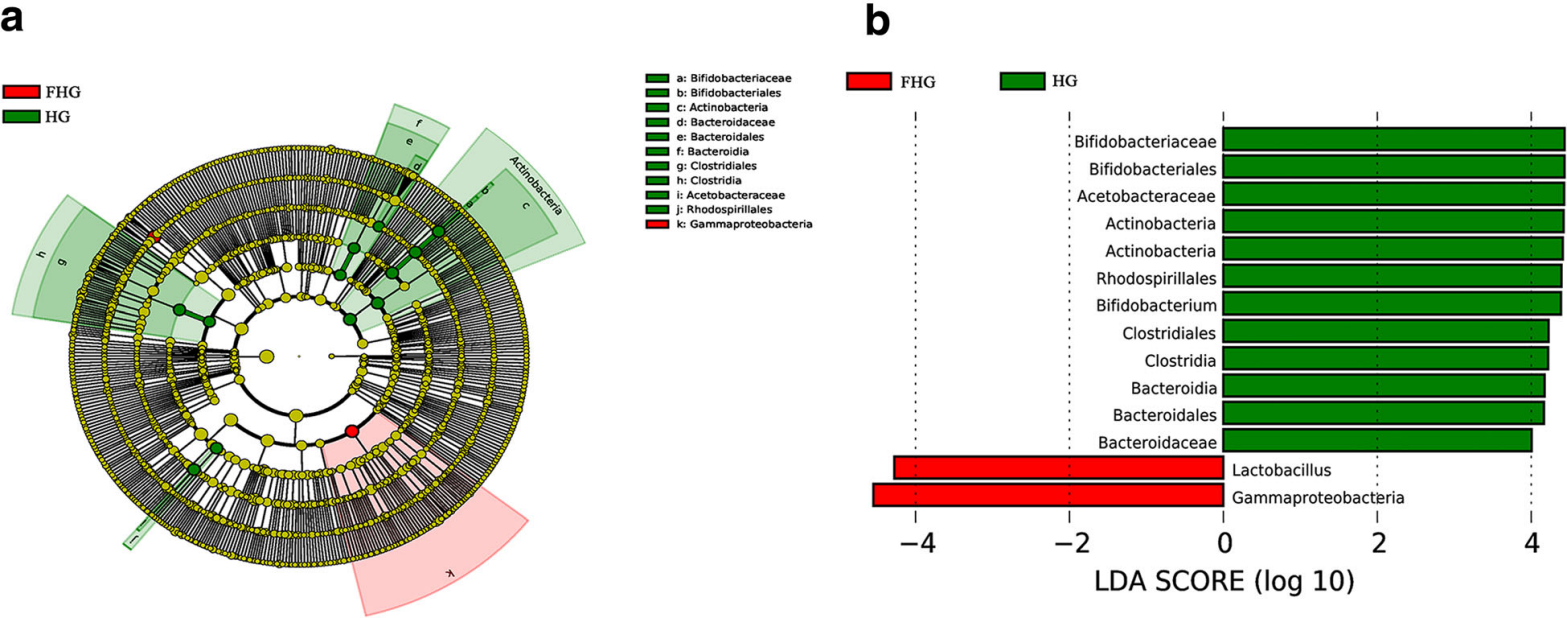
The main taxa of bacteria that were different in HG vs. FHG. **a**: Cladogram of the main taxa of bacteria that were different on the basis of LEfSe analysis. **b**: LEfSe analysis (taxa with LDA score > 4). Color code: Green represents significantly different taxa, with their highest relative abundance in HG; Red represents significantly different taxa, with their highest relative abundance in FHG

**Fig. 14:**
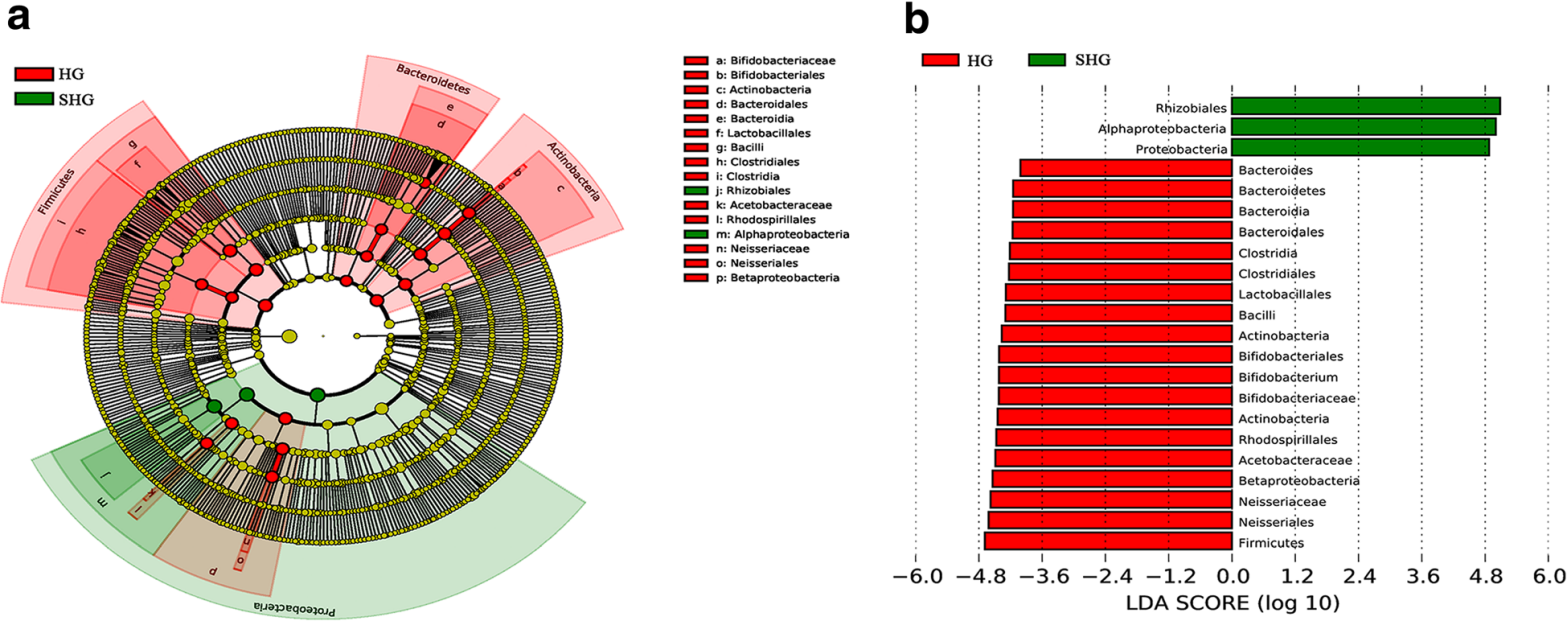
The main taxa of bacteria that were different in HG vs. SHG. **a**: Cladogram of the main taxa of bacteria that were different on the basis of LEfSe analysis. **b**: LEfSe analysis (taxa with LDA score > 4). Color code: Red represents significantly different taxa, with their highest relative abundance in HG; Green represents significantly different taxa, with their highest relative abundance in SHG

The primary microbes in hindguts that were different between prewinter and postfeeding for overwintering included Actinobacteria (*Bifidobacteriaceae*), Bacteroidia (Bacteroidales: *Bacteroidaceae*), Clostridia (Clostridiales), and Alphaproteobacteria (Rhodospirillales: *Acetobacteraceae*). The relative abundance of these taxa was higher in HG than in HHG/SHG/FHG and did not vary among the different sugars. However, there were some sugar-special effects on some microbial taxa depending on sugar type. For example, honey fed as winter food increased the relative abundance of the Pasteurellales of Gammaproteobacteria in the hindgut (Fig. 12). High-fructose syrup as winter food increased the relative abundance of Gammaproteobacteria and *Lactobacillus* in the hindgut (Fig. 13). With the exception of the above different microbes between HG and SHG, the other taxa that were different between HG and SHG included the Bacilli (Lactobacillales), Betaproteobacteria (*Neisseriaceae*), and Alphaproteobacteria (Rhizobiales). The relative abundance of Alphaproteobacteria (Rhizobiales) was higher in SHG, whereas that of the others was higher in HG (Fig. 14).

## Discussion

During overwintering, honeybees cannot obtain food from the outside world and can only consume stored sugars to produce the energy necessary to maintain colony temperature and basic metabolism. This research revealed that the type of overwintering sugar affected the microbiota of the honeybee gut. Gut microbes of honeybee have been proven to play an important role in growth, health, food metabolism, hormone regulation, improved immunity, resistance of pathogen invasion and other aspects [17–19]. Therefore, we believe that the type of sugar fed in the overwintering period will affect the health and overwintering ability of honeybees by regulating the intestinal microbiota.

The dominant microbiota established in the guts of honeybees were Proteobacteria, Firmicutes (*Lactobacillus*), Actinobacteria (*Bifidobacterium*), and Bacteroidetes. These taxa are consistent with those found in a recent study [10, 15, 16, 20–22]. These studies suggest that the dominant groups of bacteria in honeybee guts may not be easily changed by external factors, including season, temperature, sugar types, and even toxins [21]. However, in the current experiment, the relative abundance of the dominant microbiota changed with the different sugar types and seasons. For example, the relative abundance of *Lactobacillus* and *Bifidobacterium* in the hindgut was significantly higher than that in the midgut before overwintering and after bees were fed the high-fructose syrup. However, no differences were found between the honey and sucrose groups. This result may be related to the composition of food. Before overwintering, honeybees collect pollen, nectar, and other materials from the outside world. Thus, the composition of the food is complex, which may include indigestible polysaccharides and proteins that linger in honeybee guts. The market high-fructose syrup also inevitably contained some polysaccharides and proteins that were difficult to digest, which led to more feces in the hindgut. *Lactobacillus* and *Bifidobacterium* can produce pectin-degrading enzymes, glycoside hydrolases, and polysaccharide lyases, which can degrade complex carbohydrates [3, 23–26]. Thus, with higher abundance in the hindgut, *Lactobacillus* could ferment this indigestible nutrition and restrain the feces from rancidness and provide micro amounts of absorbable molecular nutrition. Therefore, the changes in microbiota abundance can function as an intestinal self-protection and self-regulation mechanism to cope with varied dietary components. This mechanism would be advantageous for honeybees to adapt to a changing environment.

Recent studies show that lactic acid bacteria (*Bifidobacterium* is also considered as a lactic acid bacterium [27]) and acetic acid bacteria are common in the honeybee intestine [28–30]. Lactic acid bacteria can increase honeybee immunity and protect the host from pathogens, bacteria, and yeast [31], and acetic acid bacteria also have potentially beneficial effects for the host [29]. When the abundance of lactic acid and acetic acid bacteria is low, host susceptibility to disease can increase [5]. In addition, treatment with lactic, formic, and acetic acids is widely employed by beekeepers to guard against honeybee pathogens [28], indicating that these bacteria are vital in protecting honeybees against pathogens. In the current experiment, an overwintering diet of sucrose increased the relative abundance of Actinobacteria (Bifidobacteriales: *Bifidobacteriaceae*) and Alphaproteobacteria in the midgut compared with the diets of honey and high-fructose syrup. Compared with prewinter, sucrose also increased the relative abundance of Actinobacteria, Lactobacillales (*Lactobacillaceae*), and Alphaproteobacteria (*Xanthobacteraceae* and *Mitochondria*), most of which are beneficial bacteria, and decreased the relative abundance of Betaproteobacteria and Gammaproteobacteria. Recent research suggests that gut microbes may have something to do with the lifespan of bees, the guts of short-lived worker phenotypes are progressively dominated by Gamma, Beta and Alpha Proteobacteria, but these same species were sparse or significantly depleted in long-lived queen phenotypes [32]. Compared with worker bee, the dominant taxa of queen were Alphaproteobacteria *Acetobacteraceae* (Alpha2.1and Alpha2.2) and *Lactobacillaceae* [33, 34]. Those researches suggested that high abundance of *Lactobacillus, Bifidobacteria*, *Acetobacter*, and low abundance of beta and Gamma may be characteristic of long-lived bee gut microbes. This is similar to the intestinal microbes composition of sucrose group. It was also found that the overwintering loss of colonies in sucrose group was lowest compared honey and high-fructose syrup (Table S4). These results suggested that sucrose, as an overwintering food, maybe prolong the lifespan of the overwintering bee by regulating intestinal microbes composition.

In addition to defense against pathogens, intestinal bacteria also play an important role in nutrition, digestion, and absorption, which can affect the growth and health of hosts [35, 36]. Intestinal symbiotic bacteria can provide nutrients to hosts, such as amino acids, vitamin B, and sterols [37], and also participate in substance metabolism and synthesis [38]. Alpha-1 (Alphaproteobacteria) are symbiotic bacteria of honeybees and may synthesize vitamins [23]. Moreover, lactic acid bacteria can convert pollen into beebread, which contains vitamins and abundant amino acids [39–41]. *Gilliamella*, *Lactobacillus*, and *Bifidobacterium*, identified as symbiotic bacteria in the gut of honeybees, produce pectin-degrading enzymes, glycoside hydrolases, and polysaccharide lyases, among other enzymes, which can degrade carbohydrates [3, 23, 24]. The effects of bacteria on host nutrition in previous studies and the presence of the supernal Alphaproteobacteria (*Acetobacteraceae*), *Bifidobacterium*, and *Lactobacillaceae* in the gut of honeybees fed sucrose in the current study both indicate that sucrose is very suitable as the overwintering food for honeybees. In addition, sucrose was more salutary and safer and cheaper than honey.

Compared with postfeeding overwintering honeybees, the relative abundance of Bacteroidetes, Clostridia (*Lachnospiraceae* and *Ruminococcaeae*), and Pasteurellales in the midgut and Actinobacteria (*Bifidobacteriaceae*), Bacteroidia (Bacteroidales: *Bacteroidaceae*), Clostridia (Clostridiales), and Alphaproteobacteria (Rhodospirillales: *Acetobacteraceae*) in the hindgut was higher in prewinter honeybees. Beneficial bacteria are found in most of these abundant taxa. For example, many members of *Lachnospiraceae* in Clostridia can produce butyric acid [42] which prevents the growth of some microbes within the digestive tract [43] and provides a source of energy for other microbes [44] and host epithelial cells [45]. Actinobacteria have been revealed as widespread symbionts of eukaryotes, their cellulolytic enzymes can efficiently break down plant biomass into simple sugars [46, 47], which can provide nutrients to the host, and producing natural products (antibiotic) to ward off pathogens and parasites [48, 49]. And in particular, the advantage of the *Bifidobacteriaceae* to host health is well known [50–52] Acetic acid bacteria also have potentially beneficial effects to the host [29]. These abundant beneficial bacteria in honeybee guts prewinter may be associated with the complex composition of the diet, in addition to the frequent activities outside the hive in prewinter, which increase the odds of interaction between honeybees and external factors, including foods, beneficial bacteria, and pathogens, among others. Thus, the abundant bacterial taxa in prewinter may contribute to the digestion of complex foods and to the resistance to pathogen attacks.

## Conclusion

The type of sugar used as winter food affected the relative abundance of the dominant bacterial communities in honeybee guts, not the taxa, which could affect the health and safety of honeybee colonies during overwintering. The presence of the supernal Alphaproteobacteria (*Acetobacteraceae*), *Bifidobacterium*, and *Lactobacillaceae* in the gut of honeybees fed sucrose and cheaper than honey both indicate that sucrose is very suitable as the overwintering food for honeybees.

## Methods

### Feeding treatment and honeybee sampling

Honeybees colonies were kept in the apiary of Shandong Agricultural University (located in east longitude 116° 20ˊ, northern latitude 35° 38ˊ). The overwintering of honeybee colonies in this area usually begins in early November. Nine overwintering colonies of honeybees (*Apis mellifera L.*) with the same colony population were randomly divided into three groups, with each including three colonies. Taking away all comb honey in the hive and respectively feeding honey (derived from the hive itself), sucrose solution (sucrose: water = 2:1) (Dehong Yingmao sugar industry Co., Ltd., Yunnan, China), or high-fructose syrup (Luzhou biological technology Co., Ltd., Linyi, China, 42% fructose) from November 2nd to 15th of 2017 as winter food. Adding 1.5 kg honey/sucrose/ high-fructose syrup to feed box in the hive every evening until the honeybees stopped moving the food to the comb (November 15th, 2017). This suggests that the bees have stored enough food to survive the winter.

Before feeding, 50 worker bees from each colony (a total of 150 honeybees per group) were collected as prewinter control samples (November 2nd, 2017). After feeding, 50 worker bees from each colony (a total of 150 bees per group) were collected on January 2nd of the next year to analyze the effects of the different sugar diets on gut microbiota.

### Gut isolation and DNA extraction

Honeybees were anesthetized on ice and washed in 75% ethanol before dissection. The head or thorax of a honeybee was fixed, and the entire intestine was removed by pulling the stinger using sterile dissecting forceps. The midgut and hindgut were separated and collected into sterile, separate microcentrifuge tubes. The total genomic DNA was extracted from samples using QIAamp 96 PowerFecal QIAcube HT kit (QIAGEN, 51531) following the manufacturer’s instructions. Then DNA concentration were verified with NanoDrop, and purity of DNA were monitored on 1% agarose gels. According to the concentration, extracted DNA was diluted to a concentration of 1 ng/μl using sterile water and stored at − 20 °C until further processing.

### High-throughput sequencing of 16S rRNA gene amplicons

High-throughput sequencing technologies were used to measure the V4 region of bacterial 16S rRNA. DNA samples were sent to the Novogene Bioinformatics Technology Co., Ltd. (Beijing, China) for sequencing. PCR amplification was conducted with the bar-coded primer pair 515f/806r (515F: GTGCCAGCMGCCGCGGTAA, 806R: GGACTACHVGGGTWTCTAAT). All PCR reactions were conducted in a 30 μL reaction volume with 15 μL of Phusion® High-Fidelity PCR Master Mix (NEB, Beijing, China), 0.2 μM forward and reverse primers, and approximately 10 ng of template DNA. The thermal cycling consisted of initial denaturation at 98 °C for 1 min, followed by 30 cycles of denaturation at 98 °C for 10 s, annealing at 50 °C for 30 s, and elongation at 72 °C for 60 s. The program was completed at 72 °C for 5 min. The same volume of loading buffer was mixed with PCR products, and electrophoresis on 2% agarose gel was performed for detection. The samples with a bright main band at 400–450 bp were chosen for further experiments. The PCR products were mixed in equidense ratios. The mixture of PCR products was purified using a GeneJET Gel Extraction Kit (Thermo Scientific, Shanghai, China). The sequencing libraries were generated using an NEB Next® Ultra™ DNA Library Prep Kit for Illumina (NEB, Beijing, China) following the manufacturer’s recommendations, and index codes were added. Library quality was assessed using a Qubit@ 2.0 Fluorometer (Thermo Scientific, Shanghai, China) and an Agilent Bioanalyzer2100 system. The library was sequenced on an IlluminaHiSeq2500 platform, and 250 bp paired-end reads were generated.

### Sequence analysis

16S RNA (V3-V4 variable regions) of gut microbiota was sequenced using a paired-end Illumina HiSeq platform to generate 250 bp paired-end reads, and the raw reads were pretreated. The specific processing steps were according to the methods reported by Manuel [53]. In brief, Paired-end reads were merged using FLASH (V1.2.7, http://ccb.jhu.edu/software/FLASH/) [54] to obtain raw tags. Noisy raw tag sequences were filtered by QIIME (V1.7.0, http://qiime.org/index.html) [55] to produce high-quality clean tags. The tags were compared to the reference database (Gold database) using the UCHIME algorithm to detect chimeric sequences. Chimeric tags were removed to obtain effective tags used for further analysis.

### Taxonomic analyses

The effective tags of all samples were clustered were clustered and classified into OTUs (operational taxonomic units) at an identity threshold of 97% similarity by using UPARSE software (UPARSEv7.0.1001, http://drive5.com/uparse/) [56]. And then the representative sequences of OTUs groups was used to assign the taxonomic category by using the SILVA database [57, 58]. For each sample, the abundance was determined for taxa at the phylum, class, order, family, and genus levels. All analyses from clustering to determining alpha and beta diversity were performed with QIIME (V1.7.0) [55] and R software (V2.15.3) was used to analyze Beta diversity index differences among groups. (Pairwise comparison using T test and multiple comparisons with Tukey test). Biomarker features were screened using Metastats (version 1.0) and LEfSe as described by Li [59].

## Supplementary information

**Additional file 1: Figure S1.** Rarefaction Curves of OTUs sampling depth.

**Additional file 2: Figure S2.** OTU abundances and taxonomic classifications within each group at different levels.

**Additional file 3: Table S1.** Data processing and quality control table.

**Additional file 4: Table S2.** Beta-diversity analysis among different groups, respectively.

**Additional file 5: Table S3.** OTU abundances and taxonomic classifications within each sample at different levels.

**Additional file 6: Table S4.** The overwintering loss of honeybee colonies fed with honey, sucrose and high-fructose syrup.

## Data Availability

All data generated or analyzed during this study are included in this published article and its supplementary information files.

## Abbreviations

FHG: Hindgut sample after bees were fed high-fructose syrup as the overwintering food
FMG: Midgut sample after bees were fed high-fructose syrup as the overwintering food
HG: hindgut sample from prewinter honeybees
HHG: Hindgut sample after bees were fed honey as the overwintering food
HMG: Midgut sample after bees were fed honey as the overwintering food
MG: Midgut sample from prewinter honeybees
OTU: Operational taxonomic unit
SHG: Hindgut sample after bees were fed sucrose as the overwintering food
SMG: Midgut sample after bees were fed sucrose as the overwintering food
