# Supplementary figures and images for: The different dietary sugars modulate the composition of the gut microbiota in honeybee during overwintering
Source: BMC Microbiol. 2020 Mar 17;20:61. doi: 10.1186/s12866-020-01726-6 (PMC7076957; doi:10.1186/s12866-020-01726-6)

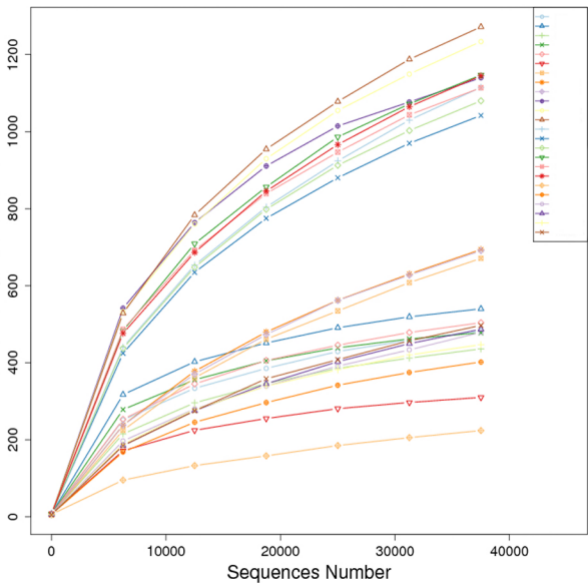

Supplement: Supplementary file 1 — Additional file 1: Figure S1. Rarefaction Curves of OTUs sampling depth. [file 12866_2020_1726_MOESM1_ESM.pdf]

**A**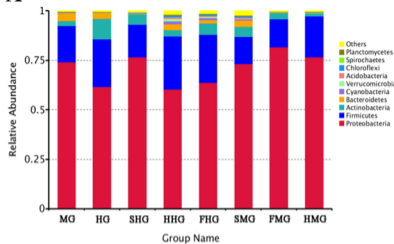**B**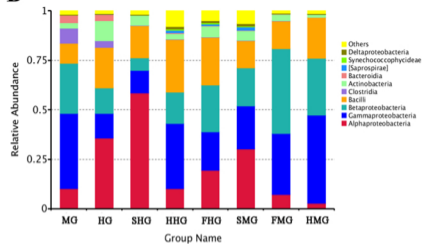**C**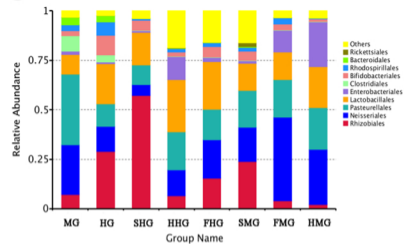**D**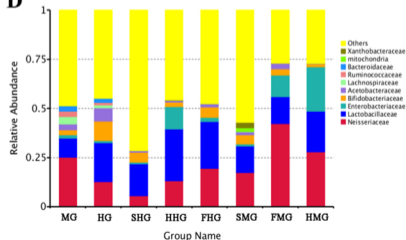**E**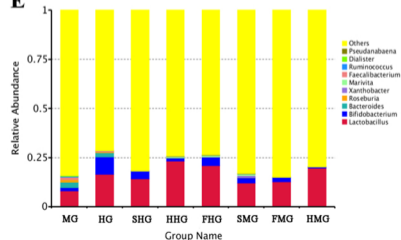

Supplement: Supplementary file 2 — Additional file 2: Figure S2. OTU abundances and taxonomic classifications within each group at different levels. [file 12866_2020_1726_MOESM2_ESM.pdf]
