# Supplementary material for: The different dietary sugars modulate the composition of the gut microbiota in honeybee during overwintering
Source: BMC Microbiol. 2020 Mar 17;20:61. doi: 10.1186/s12866-020-01726-6 (PMC7076957; doi:10.1186/s12866-020-01726-6)
Supplement: Supplementary file 3 — Additional file 3: Table S1. Data processing and quality control table. [file 12866_2020_1726_MOESM3_ESM.docx]

Table S1 Data processing and quality control table

| Sample Raw Raw Clean Effective Base AvgLen Effective Name PE(#) Tags(#) Tags(#) Tags(#) (nt) (nt) % |
| --- |
| MG1 71,197 70,444 69,441 67,994 17,286,924 254 95.50  MG2 66,982 66,128 65,162 63,543 16,127,789 254 94.87  MG3 66,561 65,768 64,792 63,635 16,164,231 254 95.60  HG1 49,868 49,305 48,580 47,602 12,049,676 253 95.46  HG2 77,481 76,731 75,843 73,650 18,627,179 253 95.06  HG3 72,308 71,633 70,754 69,679 17,631,625 253 96.36  SHG1 77,547 76,783 76,332 75,567 19,126,527 253 97.45  SHG2 72,611 71,847 71,387 70,714 17,898,818 253 97.39  SHG2 72,260 71,337 70,878 70,181 17,760,656 253 97.12  HHG1 40,361 39,709 39,418 38,900 9,851,674 253 96.38  HHG2 61,683 60,638 60,233 59,294 15,023,563 253 96.13  HHG3 73,083 71,971 71,516 70,315 17,813,496 253 96.21  FHG1 74,097 73,179 72,630 71,624 18,135,913 253 96.66  FHG2 61,136 60,017 59,403 58,447 14,879,419 255 95.60  FHG3 61,198 60,322 59,765 58,953 14,946,148 254 96.33  SMG1 64,959 62,916 62,503 58,605 14,800,306 253 90.22  SMG2 60,450 59,332 58,910 57,834 14,644,436 253 95.67  SMG3 73,339 72,139 71,606 70,262 17,864,289 254 95.80  FMG1 66,930 66,300 65,901 65,121 16,485,628 253 97.30  FMG2 57,455 56,871 56,535 55,762 14,115,849 253 97.05  FMG3 39,434 38,893 38,537 38,000 9,681,150 255 96.36  HMG1 63,103 62,535 62,161 61,559 15,573,679 253 97.55  HMG2 46,184 45,766 45,501 44,793 11,332,790 253 96.99  HMG3 63,507 62,883 62,512 61,555 15,571,418 253 96.93 |

NOTE: Raw PE: the PE reads obtained form the sequence platform. Raw Tags: the merged tags. Clean Tags: the tags by QC. Effective Tags: the tags which removed the chimeric sequences and can used by the subsequent analysis. Base: the base number of the Effective Tags. AvgLen: the average length of the Effective Tags. Effective (%): the number of Effective Tags take up of the number of Raw PE.
