# Supplementary material for: The different dietary sugars modulate the composition of the gut microbiota in honeybee during overwintering
Source: BMC Microbiol. 2020 Mar 17;20:61. doi: 10.1186/s12866-020-01726-6 (PMC7076957; doi:10.1186/s12866-020-01726-6)
Supplement: Supplementary file 4 — Additional file 4: Table S2. Beta-diversity analysis among different groups, respectively. [file 12866_2020_1726_MOESM4_ESM.docx]

Table S2 Beta-diversity analysis among different groups, respectively.

| Groups Difference  P Sig LCL UCL |
| --- |
| FMG - HMG -6 0.2479 -16.6054 4.6054  FMG - SMG 0 1.000 -10.6054 10.6054  HMG - SMG 6 0.2479 -4.6054 16.6054  HMG - MG -0.67 0.8957 -11.2721 9.9387  FMG - MG -6.67 0.2013 -17.2721 3.9387  MG - SMG 6.67 0.2013 -3.9387 17.2721  HHG - HG 5.67 0.2740 -4.9387 16.2720  FHG - HG 2 0.6946 -8.6054 12.6054  HG - SHG 9.67 0.0712 -0.9387 20.2721  HHG - SHG 15.33 0.0074 ** 4.7279 25.9387  FHG - SHG 11.67 0.03309 * 1.0612 22.2721  FHG - HHG -3.67 0.474206 -14.2721 6.9387 |

NOTE: Difference: The mean difference. P: P value. Sig: Whether a significant, if P values < 0.05 marked *, if P values < 0.01 marked ** and if P values >0.05, no marked. LCL: Confidence interval lower limit. UCL: Upper limit of the confidence interval.
