# Supplementary material for: The different dietary sugars modulate the composition of the gut microbiota in honeybee during overwintering
Source: BMC Microbiol. 2020 Mar 17;20:61. doi: 10.1186/s12866-020-01726-6 (PMC7076957; doi:10.1186/s12866-020-01726-6)
Supplement: Supplementary file 6 — Additional file 6: Table S4. The overwintering loss of honeybee colonies fed with honey, sucrose and high-fructose syrup. [file 12866_2020_1726_MOESM6_ESM.docx]

Table S4. The overwintering loss of honeybee colonies fed with honey, sucrose and high-fructose syrup

| Dietary Sugars Winter mortality |
| --- |
| Honey 65.57+2.94  High-fructose syrup 71.25+6.57  Sucrose 63.89+7.35 |
